# Effects of One Versus Two Doses of a Multi-Ingredient Pre-Workout Supplement on Metabolic Factors and Perceived Exertion during Moderate-Intensity Running in Females

**DOI:** 10.3390/sports8040052

**Published:** 2020-04-22

**Authors:** Jamie R. Erickson, Clayton L. Camic, Andrew R. Jagim, Paige M. Pellersels, Glenn A. Wright, Shaine E. Henert, Carl Foster

**Affiliations:** 1 Exercise and Sport Science, University of Wisconsin-La Crosse, La Crosse, WI 54601, USA; ericksonjamier@gmail.com (J.R.E.); gwright@uwlax.edu (G.A.W.); cfoster@uwlax.edu (C.F.); 2 Kinesiology and Physical Education, Northern Illinois University, DeKalb, IL 60115, USA; shenert@niu.edu; 3 Sports Medicine, Mayo Clinic Health System, Onalaska, WI 54650, USA; jagim.andrew@mayo.edu; 4 Physical Therapy, Nova Southeastern University, Fort Lauderdale, FL 33328, USA; pp757@mynsu.nova.edu

**Keywords:** caffeine, substrate utilization, thermogenic, fat oxidation

## Abstract

The primary purpose of this study was to examine the acute effects of one versus two doses of a multi-ingredient pre-workout supplement on energy expenditure during moderate-intensity treadmill running. In addition, our second aim was to investigate the responses of associated metabolic factors (i.e., substrate utilization, measures of gas exchange), perceived exertion, and resting cardiovascular variables with one and two doses of the pre-workout supplement. Twelve females (mean ± SD: age = 25.3 ± 9.4 years; body mass = 61.2 ± 6.8 kg) completed three bouts of 30 min of treadmill running at 90% of their ventilatory threshold on separate days after consuming one dose of the pre-workout supplement (1-dose), two doses (2-dose), and a placebo. There were no differences among conditions for energy expenditure, fat or carbohydrate oxidation, respiratory exchange ratio, oxygen consumption, or heart rate across exercise time. The two-dose group, however, had lower (*p* = 0.036) ratings of perceived exertion (11.8 ± 1.7) than the one-dose (12.6 ± 1.7) and the placebo (12.3 ± 1.2) at the 20-min time point of exercise as well as greater resting systolic blood pressure (110 ± 10 mmHg) compared to the one-dose (106 ± 10 mmHg) and the placebo (104 ± 10 mmHg) conditions. Both the one-dose and two-dose conditions had greater increases in diastolic blood pressure compared to the placebo. Thus, our findings indicated that the present pre-workout supplement had no performance-enhancing benefits related to energy metabolism but did attenuate feelings of exertion.

## 1. Introduction

Pre-workout supplementation is a nutritional strategy that involves consuming a mixture of bioactive compounds and dietary ingredients prior to a bout of exercise for ergogenic purposes. For example, pre-workout supplements often contain beta-alanine, caffeine, citrulline, tyrosine, taurine, creatine, arginine, and carnitine as well as numerous vitamins and minerals [1]. Thus, the purported effects of pre-workout supplements generally consist of elevated metabolic rate, increased measures of anaerobic and aerobic performance, and improvements in body composition [2,3,4,5,6]. Due to the relatively brief period of time that pre-workout supplements have been available, there are limited data concerning the influence of these multi-ingredient products on their ergogenic potential and variables related to general health (e.g., resting heart rate and blood pressure).

Thermogenic agents are compounds with associated effects of increasing energy metabolism [7]. For example, caffeine is one of the most commonly used thermogenic agents that stimulates the release of catecholamines (i.e., epinephrine) and renin, thereby increasing heart rate, blood pressure, lipolysis, and plasma free fatty acid concentration [3,8,9]. It has been demonstrated that acute caffeine ingestion (5–6 mg∙kg^−1^ of body mass (BM)) increases fat oxidation [10] and energy expenditure [11,12], while reducing perceived exertion [13] during exercise. In addition, caffeine supplementation (3–6 mg·kg^−1^ of BM) has been shown to increase systolic (+17%) [8] and diastolic (+6%) [14] blood pressure, oxygen uptake ($\dot{V}O$_2_) (+5–11%) [11,12], and time to exhaustion (+30%) [15] during submaximal cycle ergometry, treadmill walking, and at rest. Collectively, the findings of these investigations [8,10,11,12,14,15] indicated that acute caffeine supplementation within a wide dosing range (3–6 mg·kg^−1^ of BM) may influence metabolic, cardiovascular, psychological, and performance variables at rest and during exercise. Furthermore, it has been suggested that the combination of caffeine with other substances (i.e., capsicum, tyrosine, *Mucuna pruriens*, green coffee bean extract, *Coleus forskohlii*, and l-carnitine) may provide a synergistic effect on each of these factors [16]. 

In addition to caffeine, other compounds are purported to increase fat mobilization and oxidation. Capsicum as well as capsinoids, analogues of capsicum with low pungency, have been studied for their possible effect on appetite suppression to aid in weight loss along with their influence on body fatness and energy metabolism (i.e., increased resting fat oxidation) in humans [17,18]. It has been demonstrated that chronic capsinoid supplementation (6 mg∙d^−1^ for 12 weeks) with a moderate caloric deficit (300–500 kcal) decreased abdominal adiposity and increased fat oxidation [18] compared to a placebo. Furthermore, capsaicin supplementation (135 mg∙d^−1^ for 12 weeks) resulted in higher fat oxidation (4.2 ± 1.1 vs. 3.5 ± 0.9 g·h^−1^) and resting energy expenditure (0.7 ± 0.5 vs. 0.2 ± 0.5 MJ·d^−1^) after a four-week, very low-energy diet compared to a placebo [17]. Tyrosine, another common ingredient found in many pre-workout supplements, is a nonessential amino acid and norepinephrine precursor that may enhance the synthesis and release of catecholamines through the sympathetic nervous system [16]. *Mucuna pruriens*, a tropical legume that contains l-DOPA, has been studied for its potential to regulate blood glucose and manage hyperglycemia by the inhibition of the enzyme alpha-glucosidase that is responsible for carbohydrate digestion and glucose absorption in the digestive tract [19,20]. The consumption of green coffee bean extract (GCE), found in green or raw coffee, may also aid in weight loss by modifying glucose tolerance and hormone secretion (i.e., glucose-dependent insulinotropic polypeptide) [21,22]. Thom [23] found that coffee enriched with a GCE-derivative—chlorogenic acid—significantly increased body fat loss (3.6 ± 0.3% vs. 0.7 ± 0.4%) in moderately overweight subjects. *Coleus forskohlii*, a plant from the Lamiaceae (mint) family, is found to contain high amounts of forskolin that has been shown to increase lipolysis and the release of free fatty acids through the elevation of cyclic adenosine monophosphate and the activation of hormone sensitive lipase through the phosphorylation of protein kinase [24]. In addition, the primary metabolic functions of l-carnitine consist of transporting long- and medium-chain fatty acids into the mitochondria for beta-oxidation and to maintain energy balance by buffering short-chain acyl groups (i.e., acetyl-CoA) [25]. The proposed benefit of l-carnitine supplementation is increased fat oxidation, though it may also enhance the breakdown of branched-chain amino acids through buffering branched-chain keto acids [25].

Caffeine and these additional substances (capsicum, tyrosine, *Mucuna pruriens*, green coffee bean extract, *Coleus forskohlii*, and l-carnitine) are combined in a popular [26] pre-workout supplement (Cellucor, Bryan, TX, USA) that appears to be marketed toward females. The purported effects of this pre-workout supplement include increased lipolysis, transport, and utilization of fatty acids. To our knowledge, no research has been conducted on the safety of this product (i.e., resting heart rate, blood pressure), the metabolic influence of these combined ingredients when administered on an acute basis, or the potential of a dose–response. In particular, the suggested use for many pre-workout supplements include ingesting one or two servings depending on subject tolerance. A recent survey by Jagim et al. [26] examined the supplementation practices of 872 pre-workout users and reported that many consume more than one dose at a time and over 35% do not follow the recommended dosing instructions. Despite this, 85% of these individuals indicated that they believe consuming pre-workout, at their regular dose, is safe [26]. In addition, there are limited data regarding the influence of pre-workout supplements at these doses on metabolic, cardiovascular, and psychological variables in female populations. Therefore, the primary purpose of this study was to examine the acute effects of one versus two doses of a multi-ingredient pre-workout supplement on energy expenditure during moderate-intensity treadmill running. In addition, our second aim was to investigate the responses of associated metabolic factors (i.e., substrate utilization, measures of gas exchange), perceived exertion, and resting cardiovascular variables with one and two doses of the pre-workout supplement. We hypothesized that two doses of the multi-ingredient pre-workout supplement would result in increased energy expenditure, fat oxidation, and cardiovascular responses, while reducing perceived exertion when compared to one dose and the placebo.

## 2. Materials and Methods

### 2.1. Study Design

**Figure 1:**
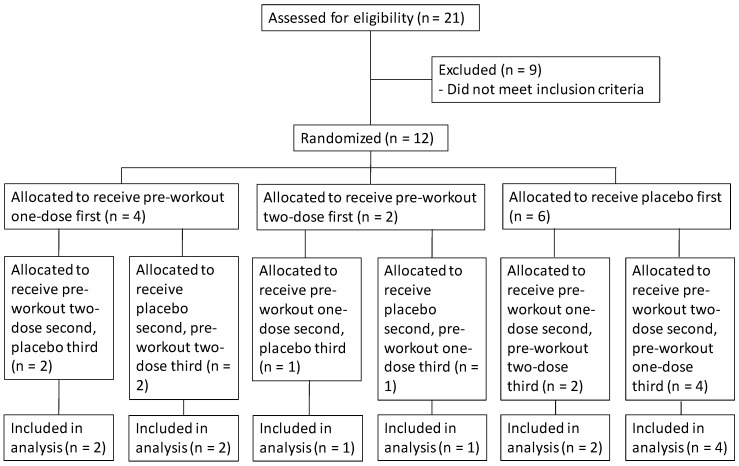
CONSORT flow diagram.

This study utilized a randomized, double-blind, within-subjects crossover design (Figure 1). Each subject was required to visit the laboratory on five occasions with 72–96 h between sessions. During the first laboratory visit, each subject performed an incremental test on a treadmill to familiarize the subjects with the testing procedures. For the second laboratory visit, each subject performed an incremental treadmill test to exhaustion to determine their ventilatory threshold (VT). The third laboratory visit was completed in the morning (06:00 a.m.–09:00 a.m.) and required subjects to consume a standardized meal after fasting overnight (8 h) and sit quietly for 30 min before baseline heart rate and blood pressure values were recorded. The subjects were then randomly assigned to ingest the supplement (one or two doses) or placebo and sit quietly for another 30 min. The ingredients of the supplement (Cellucor, Bryan, TX, USA) are provided in Table 1. The placebo was non-energetic and controlled for similar appearance and taste. At the 15-min and 30-min post-ingestion periods (of the pre-workout supplement or placebo), resting heart rate and blood pressure were recorded for a second and third time, respectively. Subjects then performed a 30-min constant-velocity treadmill run at 90% of their VT. The subjects then returned to the laboratory for their fourth and fifth visits to ingest the remaining substances (1-dose, 2-dose, or placebo) and undergo the same testing procedures (including time of day) as the third visit. Each subject recorded 2-day food logs (MyFitnessPal, Inc., Baltimore, MD, USA) prior to each laboratory visit.

### 2.2. Subjects

Twelve female subjects (mean ± SD: age = 25.3 ± 9.4 years; BM = 61.2 ± 6.8 kg) who ran ≥ 16 km·wk^−1^ were recruited to participate in this investigation. An a priori power analysis using G*Power 3.1 (Universität Düsseldorf, Germany) indicated a sample size of at least 12 was required to achieve power (1-β) of 0.80 with an effect size of 0.3 and alpha of 0.05. The subjects did not report or exhibit any of the following that could significantly affect the outcome of the study: (i) history of medical or surgical events, including cardiovascular disease, metabolic, renal, hepatic, or musculoskeletal disorders; (ii) use of any medication; (iii) use of nutritional supplements; (iv) habitual use of caffeine (≥one cup of coffee or caffeinated beverage per day); or (v) participation in another clinical trial or investigation of another investigational product within 30 days prior to screening/enrollment. All subjects were encouraged to maintain current dietary and exercise habits for the duration of the study but were asked to avoid eating and drinking anything other than water 8 h prior to laboratory visits 3–5. In addition, the subjects were asked to abstain from caffeine for at least two weeks prior to the beginning of the study. The study was approved by the Institutional Review Board for the Protection of Human Subjects (IRB title: Safety and efficacy of a multi-ingredient pre-workout supplement during low-intensity running in college-aged females; Approval Date: 24 June 2016), and all participants completed a health history questionnaire, and were informed of the benefits and risks of the investigation prior to signing an institutionally-approved written informed consent document before all testing.

### 2.3. Determination of VT and $\dot{V}O$_2peak_

During the second laboratory visit, all subjects were fitted with a heart rate monitor and informed of the peak oxygen uptake ($\dot{V}O$_2peak_) testing protocol that began at a speed of 8 km∙h^−1^ and increased 1 km∙h^−1^ every two minutes until the subject reached volitional exhaustion. Oxygen uptake ($\dot{V}O$_2_), pulmonary ventilation ($\dot{V}$_E_), carbon dioxide output ($\dot{V}\mathrm{CO}$_2_), respiratory exchange ratio (RER), and heart rate were recorded as 30-s averages throughout the test. Gas exchange was monitored using an AEI Moxus metabolic cart (AEI Technologies, Pittsburgh, PA, USA). The gas analyzers were calibrated against room air and with a certified standard mixture of oxygen (14.00%) and carbon dioxide (6.88%). The flow volume of the metabolic cart was calibrated with a 3-L calibration syringe. The test was considered maximal if the subject met at least three of the following four criteria: (a) RER was ≥ 1.1; (b) heart rate was ≥ 90% age-predicted maximum; (c) $\dot{V}O$_2_ plateaued ≤ to 150 mL·min^−1^ over the last 30 s of the test; or (d) Rating of Perceived Exertion (RPE) was ≥ 18. The subjects were verbally encouraged to exert themselves to maximal effort. The VT was determined using the method of Beaver et al. [27] via noninvasive gas exchange and was defined as the $\dot{V}O$_2_ value that corresponded to the breakpoint in the $\dot{V}$_E_ versus $\dot{V}O$_2_ relationship. Running velocities from the incremental test were plotted against $\dot{V}O$_2_ values for each subject and the regression equation derived was used to determine the running velocity that corresponded to 90% of their VT. Test-retest reliability for VT and $\dot{V}O$_2peak_ testing from our laboratory indicated the intraclass correlation coefficients (ICCs) were R = 0.93 and 0.95, and the standard error of measurements = 93 and 97 mL∙min^−1^, respectively, with no significant (*p* > 0.05) mean difference between test and re-test values. 

### 2.4. Standardized Meal and Supplementation Protocol

On visits three, four, and five, the subjects consumed a standardized meal consisting of 24% carbohydrates, 43% protein, 33% fat, and 150 kcals 60 min prior to exercise. In a double-blind manner, subjects were assigned to consume either one or two servings of the supplement (Cellucor, Bryan, TX, USA) (Table 1) or a placebo with 6 ounces of water 30 min after the consumption of the standardized meal and 30 min prior to the exercise protocol. Supplement condition order was determined through the use of a random-number generator.

### 2.5. Heart Rate and Blood Pressure

For visits 3-5, resting heart rate (HR) was recorded with a heart rate monitor (Polar Electro Inc., Lake Success, NY, USA) and resting blood pressure was measured with an automated inflatable sphygmomanometer (Omron Intelli Sense Model BP760, Lake Forrest, IL, USA) before ingesting the supplement/placebo (baseline), 15 min after ingesting the supplement/placebo (15-min post), and 30 min after ingesting the supplement/placebo (30-min post).

### 2.6. Constant Velocity Runs

During laboratory visits three, four, and five, the subjects were informed of the constant velocity run protocol and performed the test 30 min post-ingestion of the assigned supplement/placebo. This protocol consisted of the subjects running at a constant velocity of 90% VT for 30 min. $\dot{V}O$_2_, $\dot{V}$_E_, $\dot{V}\mathrm{CO}$_2_, RER, HR, RPE were recorded as 5-min averages throughout the 30-min test.

### 2.7. Estimation of Substrate Utilization

Substrate utilization was estimated by the gas exchange values recorded as 5-min averages during the constant velocity runs using the equations of Péronnet and Massicotte [28]:

$$\mathrm{Carbohydrate}\;\mathrm{oxidation}\;(g\cdot \min^{-1})=4.585\;\times \;\dot{V}{\mathrm{CO}}_{2}\;(L\cdot \min^{-1})-3.226\;\times \;{\dot{V}O}_{2}\;(L\cdot \min^{-1})$$

$$\mathrm{Fat}\;\mathrm{oxidation}\;(g\cdot \min^{-1})=1.695\;\times \;\dot{V}O_{2}\;(L\cdot \min^{-1})-1.701\;\times \;{\dot{V}\mathrm{CO}}_{2}\;(L\cdot \min^{-1})$$

Energy expenditure of non-protein RER was determined from gas exchange values by using the method of Jeukendrup and Wallis [29]:

$$\mathrm{Energy}\;\mathrm{expenditure}\;(\mathrm{kcal}\cdot \min^{-1})=0.550\;\times \;\dot{V}{\mathrm{CO}}_{2}\;(L\cdot \min^{-1})-4.471\;\times \;{\dot{V}O}_{2}\;(L\cdot \min^{-1})$$

### 2.8. Statistical Analyses

Separate two-way analysis of variance (ANOVAs) with repeated measures were used to compare energy expenditure (EE), fat and carbohydrate oxidation, RER, $\dot{V}O$_2_, RPE, and HR among the conditions (1-dose, 2-dose, placebo) at the common time points (5, 10, 15, 20, 25, 30 min) of the 30-min runs at 90% VT. In addition, separate two-way ANOVAs with repeated measures were used to compare mean resting values for HR, systolic blood pressure (SBP), and diastolic blood pressure (DBP) values among conditions (1-dose, 2-dose, placebo) at the common time points (baseline, 15-min post, 30-min post). When appropriate, follow-up tests included one-way ANOVAs with repeated measures and paired-sample t-tests with Bonferroni corrections (0.05/3 = 0.0167). Treatment order effect for each dependent variable was assessed using two-way ANOVAs with repeated measures. An alpha of *p* < 0.05 was considered statistically significant for all interaction effects and follow-up ANOVAs. In addition, separate one-way ANOVAs with repeated measures were used to compare the total caloric (kilocalories) and macronutrient (grams of protein, carbohydrate, and fat) intakes among the conditions and visits (3–5).

## 3. Results

There were no significant (*p* > 0.05) treatment order effects for any of the dependent variables measured in the present study.

### 3.1. Macronutrient Data

There were no significant differences among conditions for total kilocalories (*p* = 0.127; partial η^2^ = 0.205), fat (*p* = 0.236; partial η^2^ = 0.148), carbohydrate (*p* = 0.408; partial η^2^ = 0.095), or protein (*p* = 0.235; partial η^2^ = 0.195) consumed (Table 2).

### 3.2. Metabolic Variables

**Figure 2:**
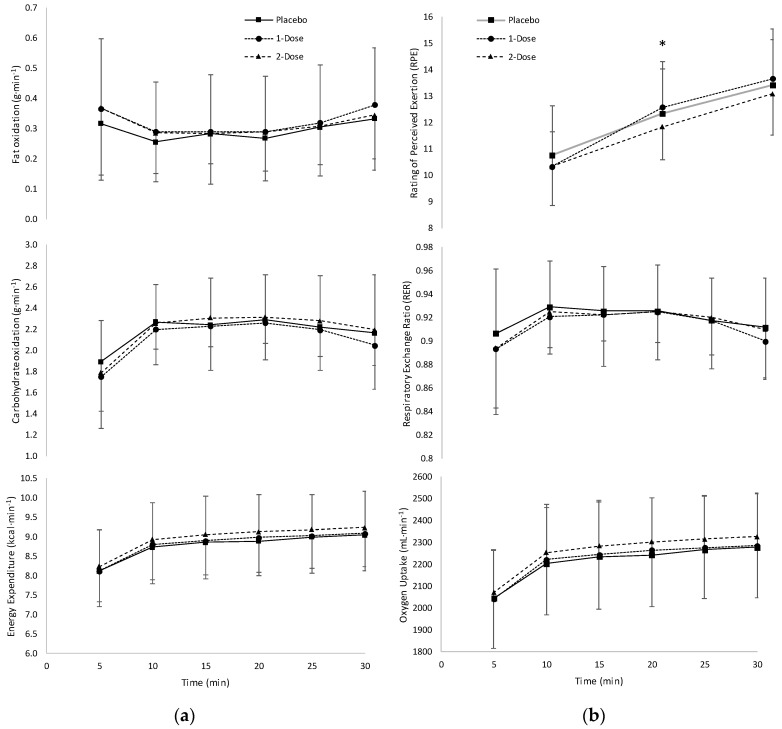
(**a**) Fat oxidation, carbohydrate oxidation, and energy expenditure values (mean ± SD) during 30 min of treadmill running for the placebo and supplement (one-dose and two-dose) conditions. (**b**) Rating of perceived exertion, respiratory exchange ratio, and oxygen uptake values (mean ± SD) during 30 min of treadmill running for the placebo and supplement (one-dose and two-dose) conditions. * Placebo and one-dose > two-dose (*p* < 0.0167).

The two-way repeated measures ANOVA for energy expenditure indicated there was no significant condition x time interaction (*p* = 0.949; partial η^2^ = 0.034) or main effect for condition (*p* = 0.138; partial η^2^ = 0.164), but there was a main effect for time (*p* < 0.001; partial η^2^ = 0.871) (Figure 2a). Values for energy expenditure across time for each condition are provided in Table 3. For fat oxidation, there was no significant condition–time interaction (*p* = 0.367; partial η^2^ = 0.091) or main effect for condition (*p* = 0.690; partial η^2^ = 0.033), but there was a main effect for time (*p* = 0.001; partial η^2^ = 0.323) (Figure 2a). For carbohydrate oxidation, there was no significant condition–time interaction (*p* = 0.237; partial η^2^ = 0.106) or main effect for condition (*p* = 0.652; partial η^2^ = 0.038), but there was a main effect for time (*p* < 0.001; partial η^2^ = 0.601) (Figure 2a). The two-way repeated measures ANOVA for RER indicated there was no significant condition–time interaction (*p* = 0.154; partial η^2^ = 0.119) or main effect for condition (*p* = 0.759; partial η^2^ = 0.025), but there was a main effect for time (*p* < 0.001; partial η^2^ = 0.401) (Figure 2b). For $\dot{V}O$_2_, there was no significant condition–time interaction (*p* = 0.907; partial η^2^ = 0.041) or main effect for condition (*p* = 0.127; partial η^2^ = 0.171), but there was a main effect for time (*p* < 0.001; partial η^2^ = 0.872) (Figure 2b).

### 3.3. Cardiovascular Variables

The two-way repeated measures ANOVA for heart rate indicated there was no significant condition–time interaction (*p* = 0.316; partial η^2^ = 0.094) or main effect for condition (*p* = 0.299; partial η^2^ = 0.104), but there was a main effect for time (*p* < 0.001; partial η^2^ = 0.984) for heart rate at rest and during exercise (Table 4). The two-way repeated measures ANOVA for SBP indicated there was no significant condition–time interaction (*p* = 0.290; partial η^2^ = 0.102) or main effect for time (*p* = 0.124; partial η^2^ = 0.158), but there was a main effect for condition (*p* = 0.002; partial η^2^ = 0.445) (Table 5). The marginal mean averaged across time for SBP was significantly greater in the two-dose condition (110 ± 10 mmHg) compared to the one-dose (106 ± 10 mmHg) and placebo conditions (104 ± 10 mmHg). For DBP, there was a significant condition–time interaction (*p* = 0.024; partial η^2^ = 0.193) (Table 5). The follow-up one-way repeated measures ANOVAs and paired sample t-tests indicated that the changes (i.e., delta scores) in DBP from PRE to 15-min post ingestion (*p* = 0.160) were not significantly different among conditions. From PRE to 30-min post ingestion, however, the two-dose (+9 mmHg; 95% CI (6,11)) and one-dose (+11 mmHg; 95% CI (6,17)) conditions exhibited significantly greater increases in DBP compared to the placebo (+3 mmHg; 95% CI (1,6)).

### 3.4. Rating of Perceived Exertion

The two-way repeated measures ANOVA for RPE indicated there was a significant condition–time interaction (*p* = 0.028; partial η^2^ = 0.215). The follow-up one-way repeated measures ANOVAs and paired sample t-tests indicated that the two-dose condition had significantly lower (*p* = 0.036) RPE (11.8 ± 1.7) than the placebo (12.3 ± 1.2) and one-dose (12.6 ± 1.7) conditions at the 20-min time point, but not the 10 or 30-min time points (Figure 2b).

## 4. Discussion

Our findings showed no changes in fat oxidation, carbohydrate oxidation, or EE among the supplement conditions and placebo at any time point during the 30-min run at 90% VT (Figure 2a). Many researchers [10,11,12,16,30,31] that have examined the effects of caffeine and caffeine-containing supplements on substrate utilization and EE reported various findings. For example, it has been demonstrated [16] that caffeine supplementation (50 mg) provided a positive thermogenic response (72 ± 25 kJ per 4 h) versus the placebo condition and increased resting metabolic rate (6% above the basal rate baseline). In addition, Hoffman et al. [30] investigated the effects of a caffeine-containing supplement on resting EE and found increases 1–3 h post-supplementation compared to the placebo group (hour 1: 1.3 ± 0.4 vs. 1.0 ± 0.3 kcal·min^−1^, hour 2: 1.3 ± 0.3 vs. 1.0 ± 0.4 kcal·min^−1^, hour 3: 1.3 ± 0.3 vs. 1.1 ± 0.4 kcal·min^−1^). Similar to our findings, Ahrens et al. [11] reported that a caffeine dose (3 mg∙kg^−1^ of BM) had no effect on EE in women at rest or during exercise. Wallman et al. [12], however, found that there was a significant increase in EE (304 ± 296 vs. 322 ± 315 kJ) at the end of 15-min cycling bout at 65% of age-predicted maximal heart rate after caffeine supplementation (6 mg∙kg^−1^ of BM (371 mg)) in sedentary females. Bergstrom et al. [31] also reported an increase in post exercise EE (5%–7%) after supplementing with a pre-workout supplement containing 200 mg (3.1 mg∙kg^−1^ of BM) of caffeine. Furthermore, Schubert et al. [10] examined the influence of caffeine (6 mg∙kg^−1^ of BM) ingestion 90 min prior to exercise on energy expenditure as well as during an hour of cycling at 65% $\dot{V}O$_2_max. The authors [10] reported that the caffeine supplement resulted in significantly greater resting EE 30 min post-supplementation (420 ± 99 vs. 361 ± 78 kJ), during exercise (3390 ± 673 vs. 3296 ± 604 kJ), and 2 h post exercise (517 ± 90 vs. 438 ± 73 kJ) compared to a control. Regarding substrate utilization, this study [10] found that fat oxidation increased 60 min prior to exercise (5.4 ± 1.4 vs. 4.1 ± 1.0 g), during exercise (30.4 ± 9.6 vs. 24.3 ± 7.2 g), 1-h post exercise (7.4 ± 2.0 vs. 6.5 ± 2.0 g), and 2-h post exercise (6.2 ± 2.3 vs. 4.5 ± 1.1 g) compared to control. Furthermore, capsicum and *Coleus forskohlii* have been investigated in chronic studies [17,18,24,32,33] and have exhibited positive effects on energy expenditure and fat oxidation. The varying energy expenditure related responses may be interpreted as positive or negative depending on the goal. For example, an increase in energy expenditure may be perceived as a positive outcome if weight loss is the desired exercise-related adaptation, whereas a performance-minded individual may seek lower energy expenditure in order to preserve energy and improve exercise economy. Additional research, however, should examine the acute effects of these ingredients on metabolic variables, heart rate, and blood pressure.

A potential reason for the differences in findings for substrate utilization in the present study compared to other protocols [10,12,16,30,31] may be due to the duration of exercise utilized. The current protocol used a 30-min time duration to remain consistent with a typical recreational workout. Exercise of longer durations at lower intensities, however, would provide more time to mobilize more fatty acids to utilize as a fuel source. Incidences of increased EE and fat oxidation in these studies [10,12,16,30,31] were likely due to catecholamine release through caffeine stimulation [3,8,9] associated with longer duration exercise. 

The present findings also indicated that there were no differences in the responses for $\dot{V}O$_2_ or RER among the conditions at any time point (Figure 2b). The lack of changes in $\dot{V}O$_2_ and RER support the reason as to why there were no changes in fat oxidation, carbohydrate oxidation, or EE. The effect of caffeine and caffeine-containing supplements on $\dot{V}O$_2_ and RER have been investigated [11,12,14,31,34] and show various results. For example, Jung et al. [34] found that supplementing with a caffeine-containing weight loss supplement increased resting $\dot{V}O$_2_ (684 ± 376 vs. 1034 ± 584 mL/min) and RER (1.48 ± 0.67 vs. 2.79 ± 0.89) toward the end of a 30-min rest protocol. During exercise, other studies [12,31] demonstrated that caffeine supplementation (6 mg∙kg^−1^ of BM and 200 mg, respectively) increased $\dot{V}O$_2_ (4%) in women during 60 min of treadmill walking with no change in RER in either protocol. Furthermore, Wallman et al. [12] found that there was a significant increase in $\dot{V}O$_2_ (17.78 ± 3.93 vs. 15.85 ± 3.64 mL∙kg^−1^∙min^−1^) at the 15 min mark of a 15-min cycling bout at 65% of age-predicted maximal heart rate after caffeine supplementation (6 mg∙kg^−1^ of BM (371 mg)) in sedentary females, but there was no change in RER at any time point [12]. In contrast, McClaran et al. [14] saw a decrease in RER (0.89 ± 0.08 vs. 0.84 ± 0.09) and no change in $\dot{V}O$_2_ after administering caffeine (3.0 mg∙kg^−1^ BM (250 mg)) to subjects during submaximal cycle ergometry. The findings of these investigations [11,12,14,31,34] and our findings indicated that caffeine and caffeine-containing supplements at various dosages may or may not impact RER or $\dot{V}O$_2_ during submaximal exercise and at rest depending on the supplementation protocol.

An important finding of the present study was that the two-dose (11.8 ± 1.7) condition resulted in a significantly lower RPE at the 20-min time point of the treadmill run compared to the one-dose (12.6 ± 1.7) and placebo (12.3 ± 1.2) conditions (Figure 2b). There were no significant differences among conditions, however, at the 10- and 30-min time points. These findings suggested that despite no differences in metabolic data among the conditions, the two-dose condition resulted in lower subjective feelings of effort during exercise. It is likely that the reduction in RPE for the two-dose condition was attributable to the amount of caffeine (300 mg (4.9 mg·kg^−1^ of BM)), which has been shown to lower feelings of exertion. In particular, Duncan et al. [13] also reported lower RPE values with a similar dose of caffeine (5 mg·kg^−1^ of BM (380 mg) during upper body exercise. Although our protocol was only 30 min in duration, these are significant findings for potential pre-workout supplement users, who may be able to increase the duration of exercise due to lowered feelings of exertion. It is also possible that the six ingredients (tyrosine, *Mucuna pruriens*, l-carnitine, green coffee bean extract, capsicum annuum, and *Coleus forskohlii*) within proprietary blends of the present pre-workout supplement contributed to these effects.

In the present investigation, the acute ingestion of one (150 mg caffeine or 2.45 mg∙kg^−1^ of BM)) or two (300 mg caffeine or 4.9 mg∙kg^−1^ of BM)) doses of the pre-workout supplement had no significant effect on resting or exercise HR during 30 min of moderate intensity running (Table 4). Previous studies that have examined the influence of caffeine and caffeine-based pre-workout supplements have reported conflicting findings [8,15,30,31,34]. For example, Hoffman et al. [30] found increases in resting HR two hours (70.4 ± 9.4 to 74.3 ± 12.6 bpm) and three hours (70.4 ± 9.4 to 72.3 ± 9.1 bpm) post ingestion of a caffeine-based (317 mg (4.4 mg∙kg^−1^ of BM)) weight loss supplement. In contrast, Daniels et al. [8] reported that consuming caffeine (6 mg∙kg^−1^ of BM (360 mg)) did not change HR at rest or during 55 min of cycle ergometry at 65% $\dot{V}O$_2max_. McClaran et al. [14], however, found decreases in HR (4–7 bpm) during submaximal cycle ergometry after caffeine administration (3 mg∙kg^−1^ (250 mg)). Similar to our findings, Bergstrom et al. [31] found that HR did not significantly increase at rest or during 60 min of treadmill walking after administering a thermogenic supplement containing caffeine (200 mg (3.1 mg∙kg^−1^ of BM)), capsicum (100 mg), and *Mucuna pruriens* seed extract (500 mg).

The present findings also indicated that the two-dose condition led to significantly (*p* < 0.0167) greater increases in resting SBP (collapsed across time) compared to the one-dose and placebo conditions (Table 5). In addition, there were greater increases in DBP from baseline to 30-min post supplementation for the one-dose (61 ± 7 to 72 ± 6 mmHg) and two-dose (66 ± 5 to 75 ± 5 mmHg) conditions, but not the placebo (64 ± 7 to 67 ± 6 mmHg). Previous findings [8,14,30,31] on the effect of caffeine and caffeine-based pre-workout supplement on SBP and DBP have reported inconsistent results. In particular, Daniels et al. [8] demonstrated that consuming caffeine (6 mg∙kg^−1^ of BM (360 mg)) increased resting SBP (17%) 20- and 40-min post-ingestion, but did not increase DBP. In addition, McClaran et al. [14] found increases in resting SBP (116 ± 13 to 123 ± 10 mm Hg) after 30 min post-caffeine ingestion (3 mg∙kg^−1^ of BM (250 mg)). Furthermore, Hoffman et al. [30] reported increases in SBP one hour (113 ± 10 to 116 ± 8 mmHg), two hours (113 ± 10 to 121 ± 7 mmHg), and three hours (113 ± 10 to 119 ± 9 mmHg) post supplementation of a caffeine-containing (317 mg (4.4 mg∙kg^−1^ of BM)) weight loss supplement. Our findings were inconsistent, however, with Jung et al. [34] who found no significant differences in SBP or DBP after administering a caffeine-containing (284 mg) pre-workout supplement. Bergstrom et al. [31] also found no changes in SBP after administering a caffeine-containing (200 mg (3.1 mg∙kg^−1^ of BM)) thermogenic supplement at rest or during exercise, but found significant increases in DBP 15-, 30-, and 60-min post-exercise. Similarly, Cameron et al. [4] reported no changes in SBP, but significant increases in DBP, following the acute ingestion of a caffeine-based pre-workout supplement. Collectively, the findings of these studies [8,14,30,31,34] indicated that the effects of caffeine-based supplements on heart rate and blood pressure are conflicting and may be due to differences in supplementation protocol (i.e., dosage, administration timing, blend of ingredients, caffeine content), conditions examined (i.e., at rest, intensity of exercise), or prior history with caffeine.

A possible explanation for the changes in HR and blood pressure found in the present investigation may be due to the physiological mechanisms associated with caffeine, including increases in total peripheral resistance (TPR), the blocking of adenosine receptors, and augmented catecholamine release. The physiological effects of caffeine consist of increased TPR through blood vessel vasoconstriction by blocking adenosine receptors that function to dilate coronary arteries to allow for increased blood flow during exercise [35]. The blocking of adenosine receptors causes adenosine to increase within the body which stimulates the release of catecholamines (i.e., norepinephrine) [35]. Catecholamines act on beta receptors and promote the release of renin that results in an increase in blood pressure [8,9,31,35]. Other possible reasons for differences among studies may be due to individual tolerances to caffeine and intensities of exercise, thereby affecting concurrent cardiovascular responses to caffeine.

The present study was not without limitations. Although the pre-workout supplement was administered 30 min prior to exercise based on the manufacturer recommendations, caffeine has been shown to reach peak levels after 60 min of ingestion [36], which would have been at the end of the 30-min run. In addition, we chose 30 min of submaximal treadmill running at 90% VT to remain consistent with the typical exercise duration and intensity of a recreational runner. The current stage of menstrual cycle was not evaluated and may have impacted the findings for RER or RPE. Furthermore, it is uncertain if the findings of the present study can be expected in male subjects due to potential differences in caffeine metabolism between genders.

## 5. Conclusions

The findings of the present study indicated that the multi-ingredient pre-workout supplement had no effect on EE, fat or carbohydrate oxidation, $\dot{V}O$_2_, RER, or HR during 30 min of submaximal treadmill running. There were, however, significantly greater increases in resting SBP and DBP for the one- and two-dose conditions compared to the placebo. Thus, our results demonstrated that a moderate dose of caffeine combined with a thermogenic blend had no effect on metabolic rate, but causes small changes in resting cardiovascular function with improved ratings of perceived exertion during 30 min of submaximal treadmill running.

## Figures and Tables

**Table 1:** Ingredients in one serving of the supplement.

Ingredient	Amount Per Serving
Vitamin C (as Ascorbic Acid)	250 mg
Niacin (as Niacinamide)	30 mg
Vitamin B6 (as Pyridoxal-5-Phosphate)	500 mcg
Folic Acid	250 mcg
Vitamin B12 (as Methylcobalamin)	35 mcg
Calcium	22 mg
Caffeine Anhydrous	150 mg
Beta Alanine	1.6 g
Arginine AKG	1.0 g
Explosive Energy Blend	221 mg
*N*-Acetyl-l-Tyrosine	
Velvet Bean (*Mucuna pruriens*) seed	
extract (standardized for l-Dopa)	
Ripped Blend	1.0 g
l-Carnitine Tartrate	
Green Coffee bean extract (standardized for Chlorogenic Acids)	
Capsimax® Cayenne (Capsicum annuum)	
fruit extract	
*Coleus forskohlii* root extract	

**Table 2:** Two-day average (mean ± SD) for total calories and macronutrients consumed during the placebo and supplement conditions (*n* = 12) *.

Variable	Placebo	1-Dose	2-Dose
Total calories (kcals·d^−1^)	1604 ± 423	1833 ± 569	1636 ± 446
Carbohydrates (g·d^−1^)	189 ± 47	204 ± 88	180 ± 65
Fat (g·d^−1^)	67 ± 29	77 ± 28	68 ± 31
Protein (g·d^−1^)	68 ± 17	83 ± 33	80 ± 29

* There were no significant (*p* > 0.05) differences among conditions for kcals or macronutrients.

**Table 3:** Energy expenditure values (mean ± SD) across time among conditions (*n* = 12).

Time (min)	Energy Expenditure (kcal·min^−1^)		
	Placebo	1-Dose	2-Dose
5	8.12 ± 0.80	8.13 ± 0.93	8.24 ± 0.94
10	8.72 ± 0.83	8.81 ± 1.02	8.93 ± 0.95
15	8.86 ± 0.83	8.90 ± 0.98	9.05 ± 0.98
20	8.89 ± 0.80	8.98 ± 0.97	9.13 ± 0.95
25	8.99 ± 0.80	9.03 ± 0.96	9.19 ± 0.90
30	9.04 ± 0.81	9.09 ± 0.97	9.24 ± 0.93

Note: There was no significant condition–time interaction (*p* = 0.949) or main effect for condition (*p* = 0.138) for energy expenditure during exercise.

**Table 4:** Heart rate values (mean ± SD) during rest and exercise among conditions (*n* = 12).

Time		Heart Rate (bpm)		
		Placebo	1-Dose	2-Dose
**Rest**	Baseline	67 ± 12	68 ± 12	65 ± 11
	Post-15	66 ± 12	64 ± 9	64 ± 8
	Post-30	69 ± 10	64 ± 12	66 ± 14
**Exercise**	5	150 ± 10	147 ± 12	144 ± 16
	10	159 ± 15	159 ± 16	157 ± 17
	15	164 ± 17	164 ± 17	162 ± 19
	20	167 ± 17	167 ± 18	165 ± 19
	25	169 ± 18	171 ± 18	169 ± 20
	30	172 ± 18	174 ± 19	170 ± 20

Note: There was no significant (*p* > 0.05) condition–time interaction or main effect for condition for heart rate at rest and during exercise.

**Table 5:** Resting blood pressure values (mean ± SD) among conditions (*n* = 12).

Time		Systolic Blood Pressure (mmHg)			Diastolic Blood Pressure (mmHg)		
		Placebo	1-Dose	2-Dose *	Placebo	1-Dose	2-Dose
**Rest**	Baseline	104 ± 12	102 ± 11	107 ± 11	64 ± 7	61 ± 7	66 ± 5
	Post-15	101 ± 9	104 ± 11	111 ± 12	67 ± 6	67 ± 7	74 ± 6
	Post-30	105 ± 10	110 ± 9	112 ± 10	67 ± 6	72 ± 6†	75 ± 5†

* Significant (*p* < 0.0167) main effect for condition collapsed across time (two-dose > placebo and one-dose). † Significantly (*p* < 0.0167) greater increase in diastolic blood pressure from baseline to post-30 min ingestion compared to the placebo.
